# Therapeutic radiation beam output and energy variation across clinics, technologies, and time

**DOI:** 10.1002/acm2.13945

**Published:** 2023-02-27

**Authors:** Mehran Miron Zaini, Jessica M. Fagerstrom, Edward I. Marshall, Kathryn M. Hedrick, Daniel Zaks, Hung Tran, Trevor M. Fitzgerald

**Affiliations:** ^1^ Northwest Medical Physics Center Lynnwood Washington USA

**Keywords:** analysis, electron, energy, output, photon, trends

## Abstract

Over the past several decades, a medical physics service group covering 35 clinical sites has provided routine monthly output and energy quality assurance for over 75 linear accelerators. Based on the geographical spread of these clinics and the large number of physicists involved in data acquisition, a systematic calibration procedure was established to ensure uniformity. A consistent measurement geometry and data collection technique is used across all machines for every calendar month, using a standardized set of acrylic slabs. Charge readings in acrylic phantoms are linked to AAPM's TG‐51 formalism via a parameter denoted *k*_acrylic_, used to convert raw charge readings to machine output values. Statistical analyses of energy ratios and *k*_acrylic_ values are presented. Employing the *k*_acrylic_ concept with a uniform measurement geometry of similar acrylic blocks was found to be a reproducible and simple way of referencing a calibration completed in water under reference conditions and comparing to other machines, with the ability to alert physicists of outliers.

## 1 INTRODUCTION

The American Association of Physicists in Medicine (AAPM) Task Group 51 formalism (TG‐51)^1^, ^2^ specifically requires measurements in water. Updated AAPM guidance on TG‐51 implementation^3^ reiterates the requirement for a water phantom; however, TG‐51 notes that the protocol does not preclude the use of solid phantoms for routine measurement of output so long as an appropriate transfer factor has been established via a calibration performed in water at least annually. Seuntjens et al.^4^ describe the framework of using an absorbed‐dose‐to‐water calibration coefficient to convert charge measured in a solid phantom to that in water for routine quality assurance of linear accelerators (linacs). The use of solid phantoms for external audits has previously been discussed in the literature^5^ as a valuable component in peer review.^6^

As this physics group expanded, it became necessary to create a uniform linac beam output and energy measurement system that could be employed at all clinics to ensure consistency. Because the group's physicists regularly provide cross‐coverage between sites, standardizing the monthly output and energy procedure allowed for consistency without shipping a phantom to many clinics. By sharing results in a centralized cloud‐based database for all linacs, automated anomaly checks flag data that deviates significantly from the average.

TG‐51 may be employed to measure the output of medical linear accelerators using an air‐filled chamber. Equation (3) of TG‐51 defines $D_{w}^{Q}$ as the absorbed dose to water at the reference depth (in water) when the ion chamber is absent:

(1) $$D_{w}^{Q}=M\cdot k_{Q}\cdot N_{D,w}^{60_{\mathrm{Co}}}.$$

Here, *M* is corrected for incomplete ion collection efficiency, polarity effects, ambient temperature and pressure differences from standard conditions, and the electrometer's calibration factor. The standardized quality assurance system described in this paper is based on the above AAPM TG‐51 protocol. This system uses stacks of polymethylmethacrylate (PMMA, also known as acrylic) blocks, commercially known as Perspex and Lucite, with a mass density of approximately 1.18 g/cm^3^, an electron density relative to water of approximately 1.147.^7^ The following equation is used with the acrylic phantom‐based setup:

(2) $$D_{w}^{Q}=M\cdot k_{\mathrm{acrylic}}\cdot N_{D,w}^{60_{\mathrm{Co}}}.$$

*k*_acrylic_ is a factor that embeds both the water‐to‐acrylic conversion and the change from reference conditions included in TG‐51 measurement geometry (including percentage depth dose (PDD) information), and $D_{w}^{Q}$ is defined as described above. Here, *M* is corrected for temperature and pressure at the time of measurement in the acrylic phantom. Note that for simplicity and consistency with group practice, this factor is referenced as *k*_acrylic_, but is dependent on beam quality *Q*, so could be named *k^Q^*_acrylic_. It is expected that the conversion factor *k*_acrylic_ is proportional to the average restricted collision stopping power ratios:

(3) $$k_{\mathrm{acrylic}}\propto {\left(\frac{\bar{L}}{\rho }\left(d_{\mathrm{acrylic}}\right)\right)}_{\mathrm{air}}^{\mathrm{acrylic}}/{\left(\frac{\bar{L}}{\rho }\left(d_{\mathrm{ref}}\right)\right)}_{\mathrm{air}}^{\mathrm{water}}.$$

The average restricted stopping power acrylic‐to‐air is acquired at the depth of interest in the acrylic phantom geometry, while the average restricted stopping power water‐to‐air is acquired at the depth of reference conditions. For photon spectra ranging with nominal accelerating potential ranging from 4 to 20 MV (energies of interest for this work) with an energy cutoff value of 10 keV, the values for ${\left(\bar{L}/\rho \right)}_{\mathrm{air}}^{\mathrm{acrylic}}$ range from 1.10 to 1.07, and corresponding values for ${\left(\bar{L}/\rho \right)}_{\mathrm{air}}^{\mathrm{water}}$ range from 1.13 to 1.10.^8^ Ratios with the same energy cutoff at 1.2 cm depth in acrylic (the depth of measurement in acrylic) range from 1.03 to 0.93, and at approximately *d*_ref_ in water, range from 1.07 to 1.02.^9^ It is noted that this method does not provide absolute dose at the depth of measurement. This system is a surrogate for the absorbed dose at the calibration geometry.

## 2 METHODS

**FIGURE 1:**
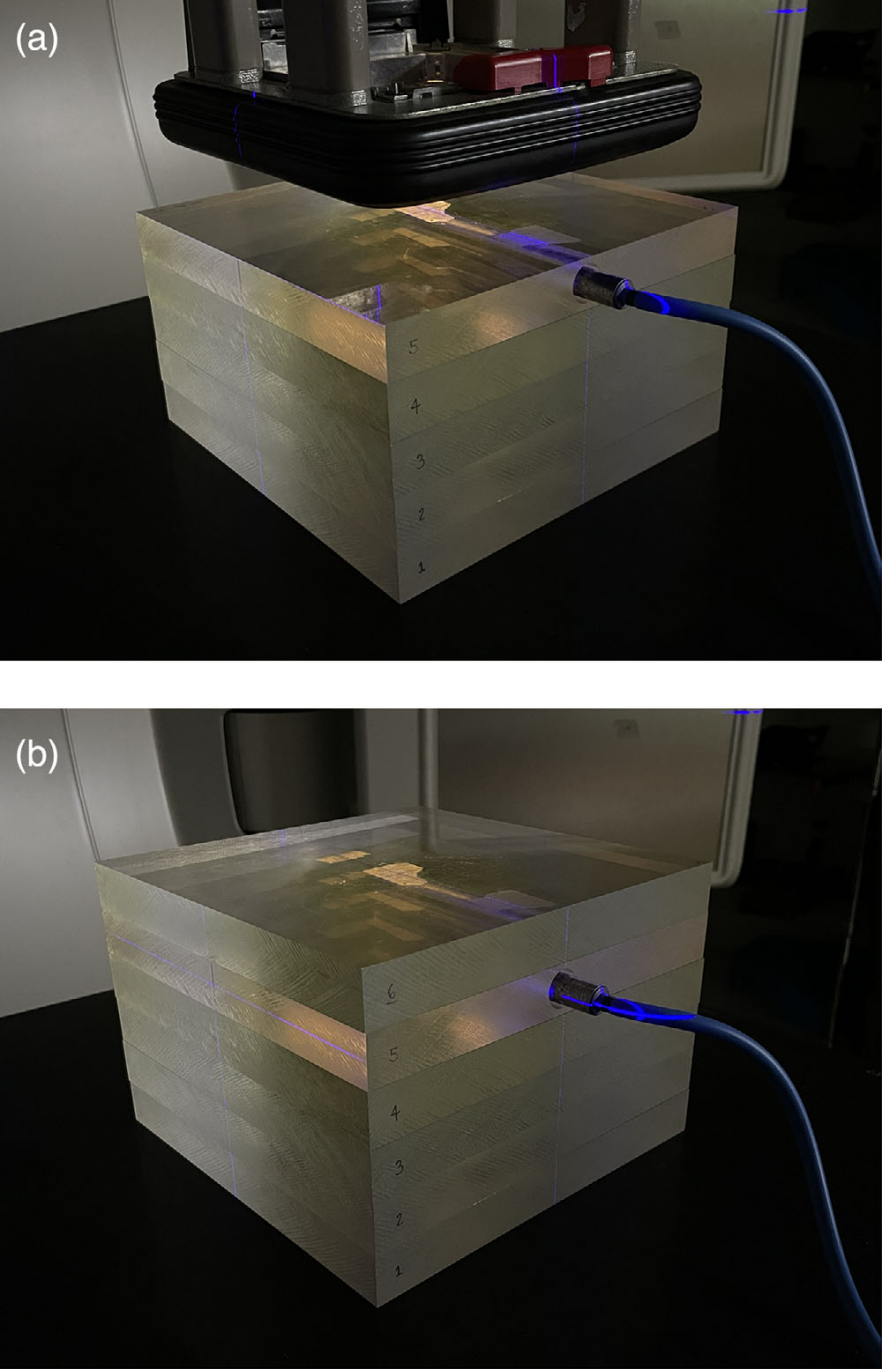
Photographs of the acrylic block phantom, with a single acrylic block drilled to accommodate a PTW 30013 farmer chamber with buildup cap. The geometry shown in (a) is arranged for electron output measurements at 100 cm SSD, with four acrylic slabs as backscatter, nominally 2.4 cm thick each, and one drilled acrylic slab. The geometry shown in (b) is arranged for photon output measurements at 100 cm SAD, with four acrylic slabs as backscatter, one drilled acrylic slab, and a sixth acrylic slab placed on top of the chamber block.

The solid phantom is composed of acrylic block slabs, each approximately (20 × 20 × 2.4) cm^3^. Slabs are purchased at nominal 1 in thickness, for which the manufacturer states an actual machined thickness of approximately 2.4 cm, with manufacturing tolerances resulting in slight variation in slab thicknesses. Because of this variation, clinics number their slabs and consistently stack the slabs in the same order for each month. One block has a hole drilled to accommodate a PTW 30013 farmer chamber with acrylic buildup cap. Four more acrylic blocks are used to provide uniform backscatter (Figure 1). For electron measurements, the approximately 1.2 cm depth of the chamber block is sufficient to avoid most of the slope of the buildup region for all energies. For photon output measurements, an additional acrylic block is used to approach the *d*_max_ depth of the photon beam. Monthly electron measurements are acquired at 100 cm source to surface distance (SSD), and photon measurements are acquired at 100 cm Source to Axis Distance (SAD).

To evaluate the constancy of each measured beam's energy, additional acrylic blocks are added so that a ratio charge measured at two standardized depths can be calculated and compared to an established baseline. For photons, three additional acrylic blocks are added to simulate a depth of approximately 10 cm. For electrons, the blocks added vary with energy such that the ratio measured is between 30% and 70% of the maximum dose, and clinics included in this analysis do not use a single, standardized phantom geometry for electron energy measurements. Therefore, electron energy results are not presented in this paper. For photon and electron output and photon beam energy verifications, the same geometry and setup is followed by all physicists at all clinics.

The *k*_acrylic_ value for each beam for each linac is established using the above materials and geometry immediately following a TG‐51 calibration in water. The ion chamber used for water measurements and the ion chamber used for acrylic measurements are not necessarily the same detector; however, they are often the same if the clinic maintains a single calibrated ion chamber. Each chamber has its own $N_{D,w}^{60_{\mathrm{Co}}}$ values, determined by ADCL calibration. The *k*_acrylic_ value is re‐established yearly at the time of the annual TG‐51 calibration or coinciding with TG‐51 calibration performed after any major linac repair. Upon initial verification of the linac's beam energy, the ratio of the reading at approximately *d*_max_ to the reading at the depth of approximately 10 cm is measured in the acrylic phantom. This ratio does not change for the life of the linac unless significant beam energy changes are intentionally made. Subsequently, the *k*_acrylic_ values and energy ratios are used to perform the monthly output and beam energy constancy checks. The *k*_acrylic_ values, energy ratios, and raw monthly readings are reported to a common database. The database is then audited as a secondary check for constancy and conformance to AAPM standards.

## 3 RESULTS

### 3.1 Linacs

The database includes 75 linacs: 57 manufactured by Varian Medical Systems, 16 produced by Elekta, and 2 by Siemens Medical Systems. These linacs reside or resided at more than 35 clinics in the United States. Beams include both conventional flattened photon beams and flattening‐filter‐free (FFF) beams. Although the uniform measurement system was adopted more than a decade ago and is still in use, the data presented here were acquired from May 2013 to May 2021. The mean duration of contiguous measurements was 1674 days (4.6 years) for all the linacs in the database. The linac with the longest continuous measurement duration spanned 3249 days (8.9 years) up until the chosen end date for the analysis.

TG‐51 describes reference conditions for photon calibration as either SSD or SAD geometry. Of the 75 linacs included in the database for the relevant period, three were SSD‐calibrated and the remainder were SAD‐calibrated. Data for the minority subset of SSD‐calibrated linacs include *k*_acrylic_ values that vary significantly from the average based on the inclusion of a factor of approximately 1/*r* ^2^. Based on the small sample size of the photon SSD‐calibrated machines, such data were removed for this analysis.

### 3.2 *k*_acrylic_ analysis

**FIGURE 2:**
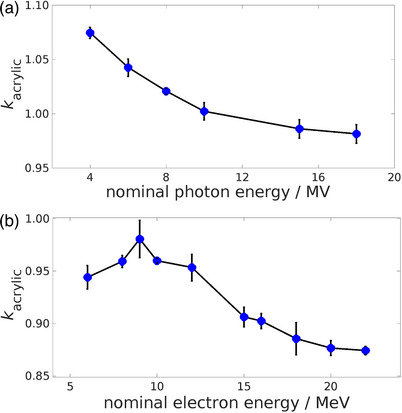
Values for normalized mean *k*_acrylic_ across all linacs. Shown in (a) are values for flattened photon beams. Shown in (b) are values for electron beams. Uncertainty bars indicate one standard deviation.

A series of 2D surface plots of all the data were generated to help identify data anomalies. This exercise served as a simple approach to data cleaning^10^ to detect and investigate potential data errors and duplicates. Photon and electron *k*_acrylic_ values for all linac types are illustrated in Figure 2.

**FIGURE 3:**
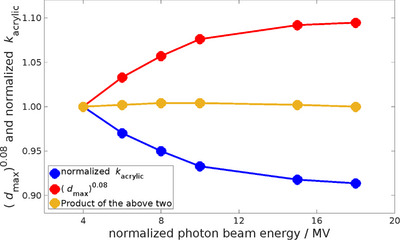
*k*_acrylic_ and *d*_max_ versus photon beam energy.

For all photon beams included in the current analysis, the depth of maximum dose measured in water is less than the measurement depth of approximately 3.6 cm in acrylic. Therefore, a conceptual correlation is expected to exist between these *k*_acrylic_ values and the *d*_max_ values. An empirical relationship was derived, comparing mean normalized *k*_acrylic_ values, in units of Gy/C, and (*d*_max_)^0.08^, in units of cm^0.08^, for each nominal photon beam energy. This relationship is summarized in Figure 3. The product of mean normalized *k*_acrylic_ values and (*d*_max_)^0.08^ is approximately unity for all beams. The observed trend underlines the correlation between the depth at which charged particle equilibrium occurs and the *k*_acrylic_ values.

### 3.3 Histograms

**FIGURE 4:**
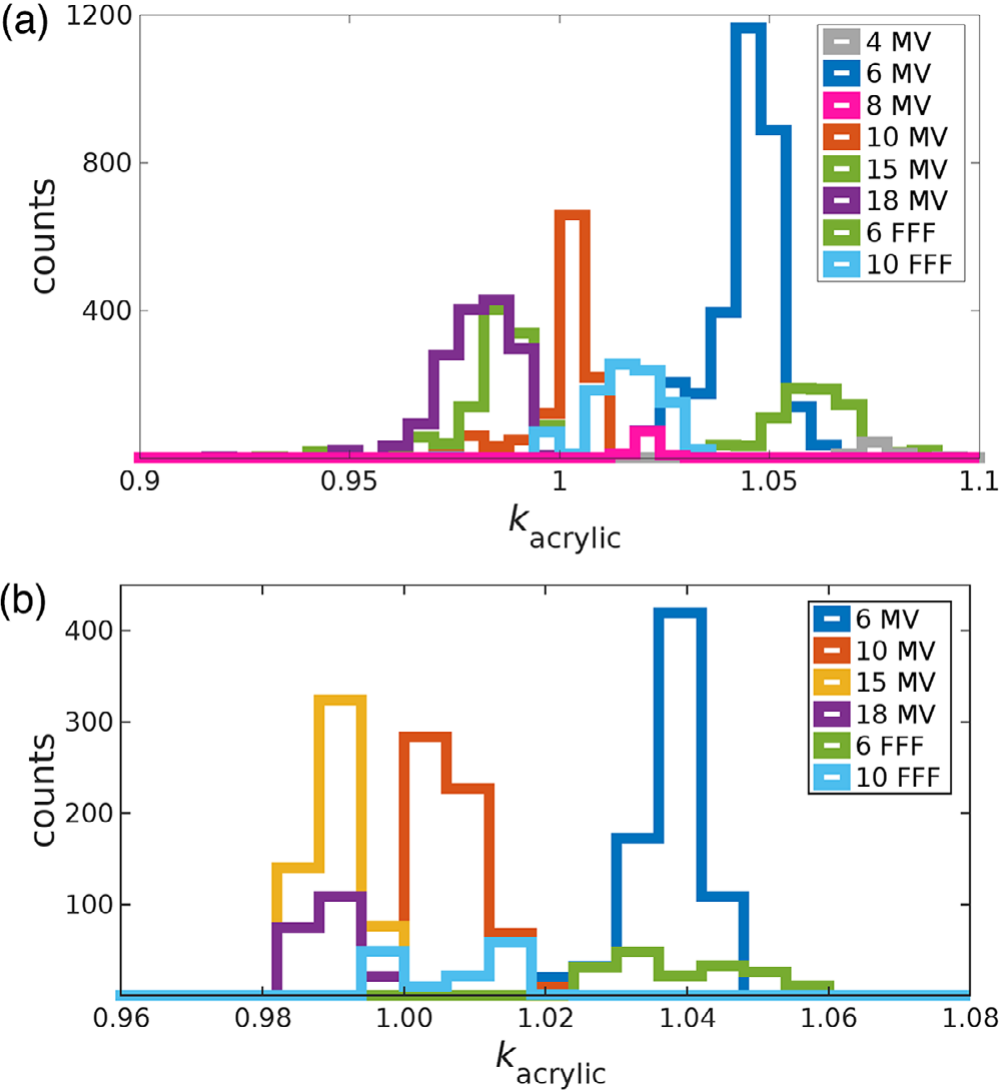
Frequency distributions of photon *k*_acrylic_ values for (a) Varian linacs and (b) Elekta linacs.

**FIGURE 5:**
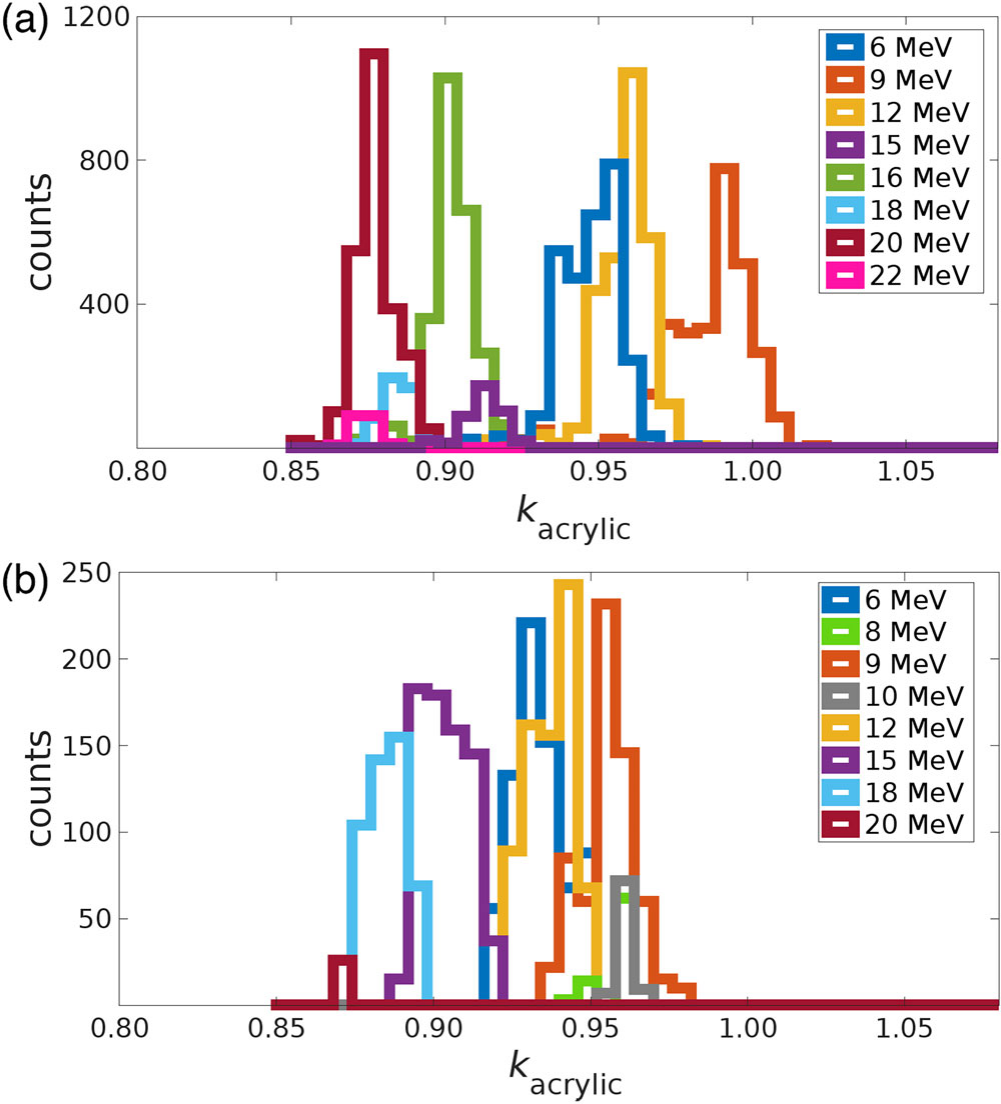
Frequency distributions of electron *k*_acrylic_ values for (a) Varian linacs and (b) Elekta linacs.

The frequency distribution of *k*_acrylic_ values was reviewed for each nominal beam energy for both Varian and Elekta linacs. Histograms of the *k*_acrylic_ values for photons beams of Varian and Elekta linacs are shown in Figure 4. Similar histograms of the *k*_acrylic_ values for electron beams are included in Figure 5.

Comparisons of the distributions of Varian and Elekta *k*_acrylic_ values were completed for nominal beam energies for which both manufacturers were represented in the dataset. For both manufacturers, *k*_acrylic_ distributions for a given nominal energy were normalized to the maximum number of counts, and then the full width at half maximum (FWHM) was acquired. As an example of this analysis, the FWHM of the normalized distribution of Varian flattened 6 MV photon beams was compared to the FWHM of Elekta flattened 6 MV, and the Varian distribution was found to be approximately 63% larger than the Elekta distribution (0.017 and 0.009, for Varian and Elekta, respectively).

### 3.4 Coefficient of variation and statistical prudence

The mean value of each beam's *k*_acrylic_ for each linac draws from a smaller sample size than the mean value of all *k*_acrylic_ values for a given energy from a given manufacturer. These smaller sets differ in size, to wit: a linac that has been in operation for a decade has many more data points than a newly commissioned machine. Sampling bias is therefore a concern. However, based on the large size of the complete dataset, the difference between the mean of *k*_acrylic_ values of a single beam from a given linac manufacturer did not differ by more than 0.3% from the mean *k*_acrylic_ value of that same nominal energy over the entire dataset of that manufacturer's linacs. Hence, the data presented here are averages of the data of the same type.

**FIGURE 6:**
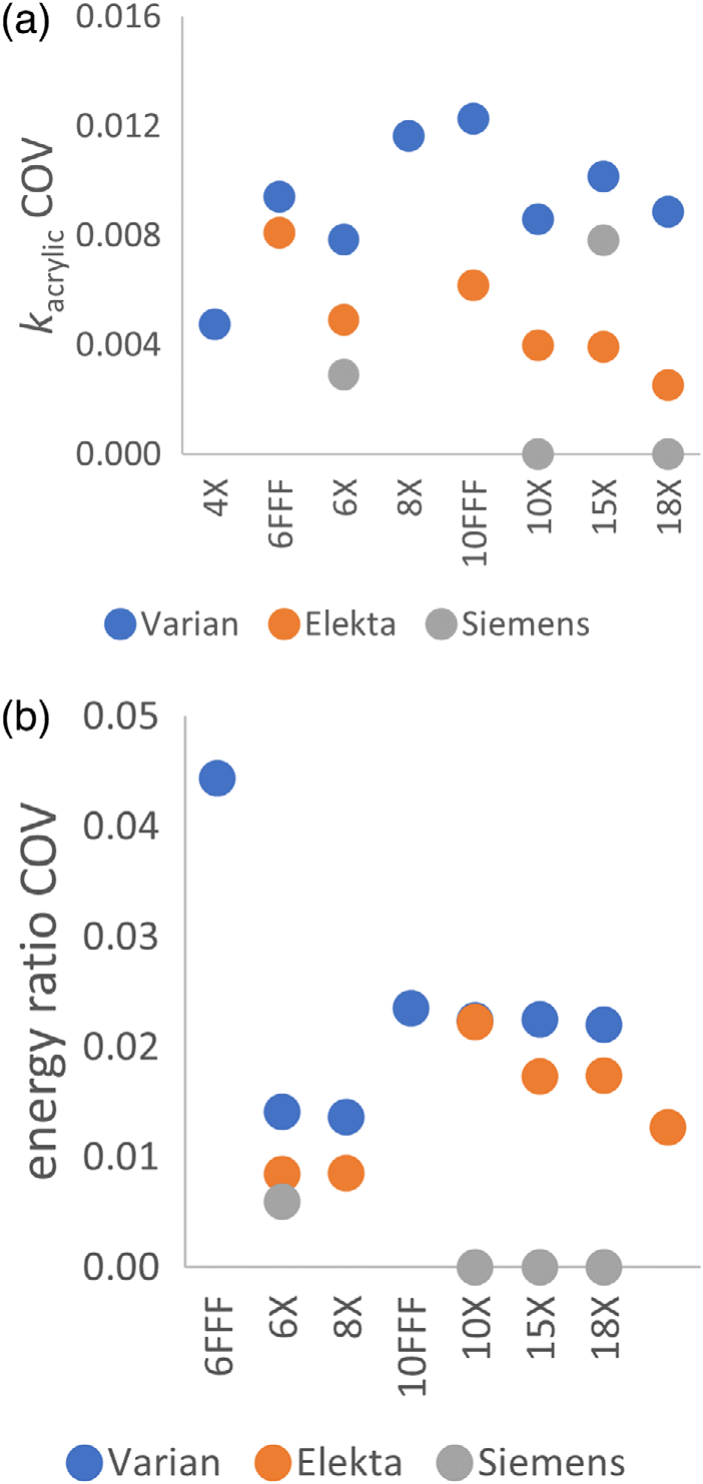
Photon coefficient of variation data for all beams of all linac types for (a) *k*_acrylic_ data, and (b) energy ratio data.

**FIGURE 7:**
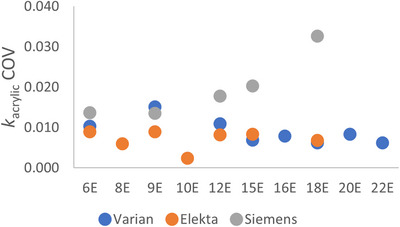
COV values calculated for *k*_acrylic_ data for electron beams for Varian, Elekta, and Siemens linacs. COV indicates coefficient of variation.

In addition to using histograms to review *k*_acrylic_ distribution data, the coefficient of variation (COV) was quantified for *k*_acrylic_ values for each nominal beam energy. COV is the ratio of the sample standard deviation to the mean, and values were calculated with Bessel's (*N*–1) correction included, where *N* is the number of data points. COV values for photon *k*_acrylic_ and energy ratio data are included in Figure 6. COV values for electron *k*_acrylic_ data are included in Figure 7.

Varian and Elekta linacs showed similar coefficients of variation (COV) for *k*_acrylic_ for all beams across different linacs at various clinics. For Varian machines, the COV of *k*_acrylic_ ranged from 0.5% to 1.5% for 16 photon and electron beams. For Elekta machines, the COV of *k*_acrylic_ ranged from 0.3% to 0.9% for 14 beams. Both Figures 6 and 7 indicate a wider variability in *k*_acrylic_ values for Varian linacs compared to those for Elekta. It is noted that the scales for Figures 6a and 7 differ by approximately a factor of 2, suggesting that the electron beams have a wider variance.

## 4 DISCUSSION

This database was designed to allow for straightforward reporting of monthly linac output and energy data, and to verify annual reference data against many other machines. The reproducibility of the set‐up geometry was planned to allow for physicists to provide cross‐coverage of different clinical sites safely and consistently, and the database provides an immediate confidence check by referencing data from many machines over several years. AAPM's TG‐100 framework^11^ discusses quality management tools, with automation and computerization (including computerized verification) listed only behind forcing functions in effectiveness. Furthermore, standardized protocols and independent double check systems (including independent review and comparison with standards) are also included in the TG‐100 framework as important tools in a quality management system. The automated flagging system for routine annual and monthly linac data may be used by other medical physics practices to support increased safety and quality.

In its current form, the database does not record direct measures of beam quality such as electron R_50_ or photon %dd(10)_X_, so beam quality information cannot be mined from the database in its current form. Future work could include revising the database submission interface so that beam quality information could later be reviewed, keeping in mind the need for simplicity and speed in uploading linac data.

Previously, the standardized procedure for linac commissioning for this group included completing a full TG‐51 calibration at the initiation of commissioning measurements to “rough in” beam output prior to running the large amount of beam required for commissioning, and then completing a final TG‐51 calibration at the conclusion of commissioning. The current analysis has demonstrated the ability to employ the *k*_acrylic_ concept (using average *k*_acrylic_ values of the appropriate linac manufacturer, machine type, and energy) instead of completing the initial TG‐51 calibration. Additionally, all database users have a powerful tool to verify that their machine and set up are within expected tolerances every time they perform a TG‐51 calibration, by comparing across linacs over time. A physicist performing TG‐51 on a machine currently in the database can use *k*_acrylic_ as an additional layer of safety—unexpected fluctuations in *k*_acrylic_ for a given beam on a given linac can act as a warning to re‐examine the machine and the TG‐51 measurement process.

Note that a full, expanded uncertainty analysis (for example, as described by NIST Technical Note 1297^12^) for *k*_acrylic_ and COV values was not performed and is considered outside the scope of this technical note. Variation in parameters outside of machine performance (such as acrylic slab thickness differences based on manufacturing tolerances) is expected to contribute to some of the variation in values across clinics. The purpose of the standardized measurement procedure and reporting process is to add a level of risk reduction, and the above analysis indicates that this framework is a useful tool.

## 5 CONCLUSION

This analysis has shown that a large medical physics service group, distributed over an extended geographical region and over 10 years, can employ a consistent methodology for routine monthly linear accelerator output and energy quality assurance. The described approach uses a standardized acrylic phantom geometry that is traceable to TG‐51 measurements under reference conditions, such that each clinical site may maintain its own relatively inexpensive monthly QA phantom. Results are maintained in a centralized data repository. Such tables include each individual linac beam's values for the transfer coefficient used to convert charge measured in the standardized phantom to dose under TG‐51 reference conditions. Results also include photon charge ratios using two different depths of acrylic buildup as a quantification of beam energy.

## AUTHOR CONTRIBUTION

**Mehran Miron** Zaini: Data Measurements, Database Generation, Mathematical Analysis, Text Preparation, Graph Creation.

**Jessica M**. Fagerstrom: Data Measurements, Data Analysis, Text Preparation, Review and Editing.

**Edward I**. Marshall: Data Measurements, System‐wide Data Collection, Database Generation, Data Organization, Text Preparation.

**Kathryn M**. Hedrick: Data Measurements, Logistics, Review.

**Daniel** Zaks: Data Measurements, Database Organization.

**Hung** Tran: Data Measurements, Database Manipulations.

**Trevor M**. Fitzgerald: Data Measurements, Program Design.

## CONFLICT OF INTEREST STATEMENT

The authors declare that there is no conflict of interest that could be perceived as prejudicing the impartiality of the research reported.
